# A Skills-Based HIV Serostatus Disclosure Intervention for Sexual Minority Men in South Africa: Protocol for Intervention Adaptation and a Pilot Randomized Controlled Trial

**DOI:** 10.2196/36845

**Published:** 2022-05-16

**Authors:** Joseph Daniels, Remco P H Peters, Andrew Medina-Marino, Cikizwa Bongo, Rob Stephenson

**Affiliations:** 1 Edson College of Nursing and Health Innovation Arizona State University Phoenix, AZ United States; 2 Foundation for Professional Development East London South Africa; 3 University of Cape Town Cape Town South Africa; 4 Desmond Tutu Health Foundation University of Cape Town East London South Africa; 5 Department of Psychiatry University of Pennsylvania Philadelphia, PA United States; 6 Department of Systems, Populations and Leadership University of Michigan School of Nursing Ann Arbor, MI United States

**Keywords:** gay, bisexual, men who have sex with men, HIV intervention adaptation, videoconference delivery, HIV, public health, mobile phone

## Abstract

**Background:**

Gay, bisexual, and other men who have sex with men (GBMSM) living with HIV have low antiretroviral treatment adherence in South Africa due to limited skills in managing disclosure and prevention behaviors with sexual and romantic partners. As a result, there is a high HIV transmission risk within HIV-discordant partnerships, but an existing intervention may address these outcomes, if adapted effectively. Healthy Relationships (HR) is a behavioral intervention that was originally delivered in groups and in person over 5 sessions to develop coping skills for managing HIV-related stress and sexually risky situations, enhance decision-making skills for HIV disclosure to partners, and establish and maintain safer sex practices with partners. HR effectively improves prevention behaviors but has yet to be tailored to a non-US context.

**Objective:**

We aim to adapt HR into a new culturally grounded intervention entitled *Speaking Out & Allying Relationships* for GBMSM and then assess its feasibility in Eastern Cape, South Africa.

**Methods:**

The study will have 2 aims. For aim 1—adaptation—we will use a human-centered design approach. Initial intervention tailoring will involve integrating Undetectable=Untransmittable and pre-exposure prophylaxis education, developing intervention content for a videoconference format, and designing role-plays and movies for skill building based on preliminary data. Afterward, interviews and surveys will be administered to GBMSM to assess intervention preferences, and a focus group will be conducted with health care providers and information technology experts to assess the intervention’s design. Finally, a usability test will be performed to determine functionality and content understanding. Participants will be GBMSM living with HIV (n=15) who are in a relationship and health care providers and information technology (n=7) experts working in HIV care and programming with this population. For aim 2, we will examine the feasibility of the adapted intervention by using a pilot randomized control design. There will be 60 individuals per arm. Feasibility surveys and interviews will be conducted with the intervention arm, and behavioral and biomedical assessments for relationship and treatment adherence outcomes will be collected for both arms. All participants will be GBMSM living with HIV who are in a relationship with an HIV-negative or unknown status partner.

**Results:**

Intervention adaptation began in August 2021. Initial tailoring and the refining of GBMSM intervention preferences were completed in December 2021. Usability and feasibility assessments are due to be completed by March 2022 and February 2024, respectively.

**Conclusions:**

GBMSM need efficacious interventions that tackle partnership dynamics, HIV prevention, and treatment outcomes for antiretroviral treatment adherence and viral suppression in South Africa. Harnessing everyday technology use for social networking (eg, videoconferences), Undetectable=Untransmittable education, and pre-exposure prophylaxis to update an existing intervention for South African GBMSM has the potential to strengthen relationship communication about HIV treatment and prevention and, in turn, improve outcomes.

**International Registered Report Identifier (IRRID):**

DERR1-10.2196/36845

## Introduction

### Background

The past 20 years have seen a growth in HIV research and programmatic attention focused toward gay, bisexual, and other men who have sex with men (GBMSM) in Southern Africa. What was long considered a generalized epidemic among heterosexual individuals is now recognized to be paralleled by a devastating epidemic among GBMSM [1]. To date, the recruitment of GBMSM for HIV surveillance and intervention studies has consistently demonstrated high uptake, retention, and acceptability in South Africa [2-5]. Moreover, these studies have reported HIV prevalence rates ranging from 13.2% to 49.5% for GBMSM, but only 10% to 18.6% of these GBMSM are taking antiretroviral treatment (ART) [3,6,7]. Interventions are needed to address this gap, especially if these consider the influence of relationships on HIV treatment outcomes.

South African GBMSM are often not adherent to their ART while in relationships because of poor skills for managing HIV disclosure and prevention with partners [8]. Among GBMSM who have access to ART, 91% are suboptimally adherent, and many do not know that optimal adherence can lead to viral suppression and the elimination of onward transmission (ie, Undetectable=Untransmittable [U=U]) [9-13]. Stigma acts as a significant barrier to ART initiation [14]. Engaging in HIV care and treatment requires men to disclose their serostatus to others, and there is evidence that social isolation and rejection by sexual or romantic partners prevent men from adopting ART [15,16]. As a result, GBMSM often drop out of clinical care after testing HIV positive, and during this time, they do not disclose their HIV status to partners or use condoms and lube, leading to an increased HIV transmission risk [2,17]. Only after developing an AIDS-related illness however do GBMSM start ART, if at all [8,18]. To prevent being involuntarily disclosed as HIV positive to their sexual partners, GBMSM will often skip ART dosages regularly or stop altogether [8,17].

In previous work, Daniels et al [19] demonstrated that GBMSM relationship dynamics encompass inabilities to discuss safe sex and limited serostatus disclosure—dynamics that result in suboptimal ART adherence. Also, in their study, a 1- to 2-year drop-off along the treatment cascade after testing HIV positive was commonly discussed, corresponding with similar findings for GBMSM in Kenya [1,17,20]. At present, GBMSM do not disclose their HIV status to other GBMSM, thereby increasing social isolation [2,15]. Further, it was shown that for each year of age that participants identified as GBMSM, their odds of testing HIV positive increased by 27%, and the coefficient for the number of clinic visits decreased by 14.5% [21]. Another major qualitative theme revealed that GBMSM will stop ART when starting a new relationship or moving in with their boyfriend out of fear of involuntary HIV disclosure, which is similar to findings in other African settings [18,19]. However, after being out of care and developing AIDS-related illnesses, 84% of GBMSM reported that they disclosed their HIV status and sexuality to an immediate family member. Disclosure allowed them to secure support for their HIV treatment, but this HIV disclosure does not extend to partners [21]. Consequently, GBMSM often encounter significant levels of HIV-related stress and exhibit poor coping and disclosure skills.

Addressing partnership dynamics may improve the effectiveness of HIV treatment interventions for GBMSM in South Africa. However, few HIV treatment interventions have been implemented with African GBMSM, and a growing body of global evidence suggests that GBMSM partnership dynamics are fueling HIV transmission such that one-third to two-thirds of new HIV infections among GBMSM occur within serodiscordant partnerships [22-24]. In South Africa, one study found high rates of regular partnerships for GBMSM (70.5%-75.7% of participants), within which HIV infection was significantly associated with reporting a primary male partner [6]. Among a large sample of male couples (N=300), the prevalence of HIV was high (42%), with 33% of men in serodiscordant relationships living in Kwa-Zulu Natal, South Africa [25]. Despite high levels of HIV testing in the past 6 months (65%), condom use with sex partners has been low (8%). Further, GBMSM have reported low levels of willingness to use pre-exposure prophylaxis (PrEP; 16%) [26]. Factors that limit engagement in HIV prevention among partnered men include relationship dynamics (ie, poor communication skills, the misplaced belief that relationships are protective of HIV, and fears of partner rejection) and stigma from health care providers [16,25]. However, these outcomes can be mediated by skill building in partner communication, disclosure, and prevention.

Healthy Relationships (HR) is a behavioral intervention that was originally delivered in groups and in person over 5 sessions. HR aims to (1) develop coping skills for managing HIV-related stress and sexually risky situations, (2) enhance decision-making skills for HIV disclosure to partners, and (3) establish and maintain safer sex practices with partners. HR integrates edited movie scenes for participants to view and role-plays that model effective communication skills [27]. HR has been used with HIV-positive GBMSM and women in the United States. In the original HR intervention, Kalichman et al [27] recruited 176 participants into the intervention arm, of whom 88% were GBMSM. Those who received the intervention showed a significant decrease in unprotected sex rates and a lower risk of HIV transmission at 6 months. Marhefka et al [28,29] adapted HR into a videoconference format, delivering the entire intervention to women in rural Florida. Participants in the intervention expressed a high satisfaction rate (84%) and had 7 fewer unprotected sex incidents compared to those among the control participants [28-30].

In our study, HR will be adapted and pilot-tested as a videoconference-delivered intervention for HIV-positive GBMSM with partners by using a design process that incorporates local voices to create an intervention that reflects the lived realities of South African GBMSM. This project aims to develop HR for GBMSM in South Africa into a new, culturally adapted intervention entitled *Speaking Out & Allying Relationships* (SOAR). SOAR will be delivered via the internet, with participants joining via smartphone and video chat. HIV treatment interventions delivered via videoconference are feasible, given the expansion of inexpensive smartphone technologies in South Africa; 84% of adults own a smartphone with which they access the internet and engage with social media [31]. Increased evidence shows that GBMSM access web-based social networking sites and use SMS text messages for social networking through mobile and smartphone technologies, creating the potential to tag on ART adherence interventions [32-34].

Web- and group-based HIV interventions are demonstrating feasibility in diverse African settings. SMART Connections is a 5-session ART adherence and retention in care intervention that was delivered to HIV-positive youth in Nigeria through closed, secret Facebook group sessions [35]. Feasibility results showed high engagement, with at least half of the participants engaging in all sessions and providing recommendations for larger groups. In South Africa, a web-based chat room was offered to youth for HIV support between adherence club sessions by using the MXit social network platform (MXit Ltd) [36]. This intervention showed mixed results; 34% of participants used the web-based group at least once, and 84% approved of the group, but there was a loss of interest. This suggested the need for an adaptation that is tailored to youth subgroups. A small-group, video-based HIV prevention intervention delivered over 5 sessions for motivational skill building, when compared to HIV education only for female military personnel in Nigeria, was effective at 3- and 6-month follow-ups [37]. Given this emerging evidence for web-based HIV interventions, adapting an evidence-based intervention into a videoconference format for GBMSM may be feasible and improve their HIV treatment outcomes.

To further support the proposed delivery method of their intervention, Daniels et al [38] found that GBMSM are willing to complete HIV-related group work, as it is empowering for GBMSM to learn that they have shared experiences based on their sexuality and HIV status. GBMSM receptivity supports the group-based design of an intervention. Further, GBMSM are interested in HIV treatment interventions that use mobile health (mHealth) tools, and videoconference modalities offer the opportunity to create the group experience that GBMSM value [39]. Specifically, Daniels et al [39] showed that mHealth interventions are feasible, given that 71% of 20- to 25-year-old GBMSM own a smartphone. In the same study, 83% reported that they would be interested in engaging in a web-based platform for combined HIV and GBMSM support. These basic feasibilities and preferences support our planned technical adaptations and enhancements for SOAR, including a videoconference format.

Herein, we present our protocol for intervention adaptation followed by a pilot randomized controlled study to assess SOAR feasibility for South African GBMSM. The findings from our study will inform a larger clinical trial for determining SOAR effectiveness in improving HIV disclosure, relationship communication, and viral suppression.

### Theoretical Framework

SOAR will be guided by social cognitive theory (SCT), which posits that cognition, behavior, and environment interact and influence health outcomes, like HIV risk reduction, disclosure, prevention, and ART adherence [28,40]. The primary focus of SCT is on self-regulation and self-efficacy [41,42]. In SCT, individuals conduct a cognitive process of determining and weighing the costs and benefits of completing expected health behaviors, such as the self-regulation of disclosure stress, that are enhanced by supportive environments (ie, intervention sessions and check-ins via videoconference) [29,42]. The intervention is expected to enhance skills for coping with HIV-related stress and build skills for the self-regulation of disclosure, treatment, and prevention [41]. These are complemented with self-efficacy, which is the ability to be motivated and have confidence in healthy behaviors and communication in relationships [43]. Within the SCT model, self-regulation and self-efficacy are facilitated by HIV risk reduction; treatment education, including U=U, HIV prevention (eg, partner referrals for HIV testing), and PrEP education; and skill building for the disclosure of HIV status and sexuality in a confidential group environment [41,43]. The ability of GBMSM to plan for safe sex and consider HIV disclosure to partners through the intervention will support ART adherence and engender a sense of agency, thus reducing the negative feelings related to their HIV status and internalized HIV stigma [2,33].

## Methods

### Ethics Approval

Our study has been reviewed and approved by the University of Cape Town Review Board (approval number: FWA00001938) with reliance by the institutional review boards at Arizona State University (approval number: STUDY00014539) and the University of Michigan (approval number: HUM00208997).

### Study Design Overview

The study will involve intervention adaptation (aim 1) followed by a randomized controlled trial (aim 2) to assess feasibility. For adaptation, we will use a human-centered design (HCD) approach. HCD is a multistep approach to gathering different perspectives and experiences from key stakeholders (GBMSM and health care and information technology [IT] specialists) and end users (GBMSM in relationships) for iterative intervention adaptation to include technology integration in context for implementation [44]. Furthermore, our HCD approach will involve initial tailoring based on preliminary research and the refining of GBMSM intervention preferences, followed by the determination of usability. Finally, a feasibility study will be conducted by using a randomized control trial design with GBMSM in relationships. Although the study will not be powered to detect behavioral changes resulting from the intervention, the findings will provide a baseline to determine the intervention effect for the subsequent clinical trial.

### Aim 1 Study Procedures for the Adaptation of HR Into SOAR

#### Initial Tailoring

HR tailoring will be completed by the research staff to include the integration of U=U and PrEP education and messaging, and based on preliminary research, contextually relevant movie segments and role-plays for coping and risk assessment skill building will be developed. Additional intervention components that will be developed include a partner referral letter for HIV testing, action plans, and a monthly videoconference group check-in. The partner referral letter will provide access to HIV testing, U=U and PrEP education, and related local services that are available in the area and be designed to be provided by the participants, who will self-report delivery only [45]. The objective of the letter is to serve as 1 of 2 measures of partner engagement in HIV prevention and care [46]. Action plans are the second measure, and they will be designed such that GBMSM can devise a plan for coping and deciding whether to disclose their HIV status to their partners. Action plans will be discussed during the monthly group check-in after the five core group sessions are completed.

#### Refining GBMSM Intervention Preferences

We will conduct 15 individual interviews with GBMSM and 2 focus group discussions with a total of 12 health care providers, program leaders, and IT experts. Interviews will be conducted with GBMSM to assess their experiences with and preferences for disclosure and their interests in and considerations for participating in an intervention that builds HIV disclosure skills and is delivered via videoconferences in a group setting. Additionally, by using the findings from the interviews, a study-specific survey will be developed to refine GBMSM participants’ preferences for the intervention. Afterward, focus groups will be conducted with health care providers, program leaders, and IT specialists who provide HIV care or manage GBMSM programming in Eastern Cape and on the internet. The focus group discussions will involve a presentation of the initially tailored HR from the initial tailoring phase, and we will seek feedback on session content based on their experiences with working with GBMSM in this setting. The findings from interviews, focus groups, and study-specific surveys will be integrated into the intervention and then assessed for usability.

#### Usability

The intervention will be pretested for usability over a 5-week period in 2-hour sessions (1 session per week). A group check-in will then be conducted 3 weeks after participants’ last session (week 8). A group of participants (n=7) will complete all 5 sessions together on Zoom (Zoom Video Communications Inc). During the last session, participants will be informed of the date and time for the group check-in session. During the pretest, the interventionist will monitor participants’ engagement and follow up with participants who miss a session. Before beginning the pretest, each participant will provide their mobile number to receive group session reminders. All intervention sessions and group check-ins will be video recorded for analysis. All participants will receive a data plan to complete this task.

After the pretest, interviews will be conducted with GBMSM participants, and a focus group will be conducted with health care and IT experts. The interviews with GBMSM participants will cover the following six intervention usability domains [47]: (1) the functionality of sessions with group check-ins, (2) the timeliness and appropriateness of sessions, (3) the clarity of session content delivery, (4) the clarity and management of self-assessments and action plans, (5) incomplete sessions, and (6) the technical transition between sessions and between sessions and group check-ins. The focus group with health experts will involve a presentation of the intervention data that are collected from the pretest. This presentation will include a video recording of 1 session, interview results, and technical challenges. Health experts will be asked to provide their perspective on how these pretest findings will influence the pilot intervention and what corrections are needed to limit the number of incomplete group sessions and technical challenges, in order to improve usability. The findings will be integrated into the final intervention and named *SOAR* for the pilot test in the randomized controlled trial (aim 2).

### Aim 1 Participants

There will be 15 HIV-positive, partnered GBMSM recruited from HIV outreach activities that are led by a local collaborating organization. Further, 12 health care providers, program managers, and IT experts who work in GBMSM programming in Eastern Cape will be purposely recruited. For the usability test, there will be 7 GBMSM participants recruited from the initial 15, and all 12 health care providers, program managers, and IT experts will be recruited. All participants will complete written informed consent and will receive R150 (around US $10) as a travel reimbursement for completing the study activities and a R150 (around US $10) data plan to support their session attendance.

### Aim 2 Study Procedures for the Feasibility Assessment of SOAR

**Figure 1:**
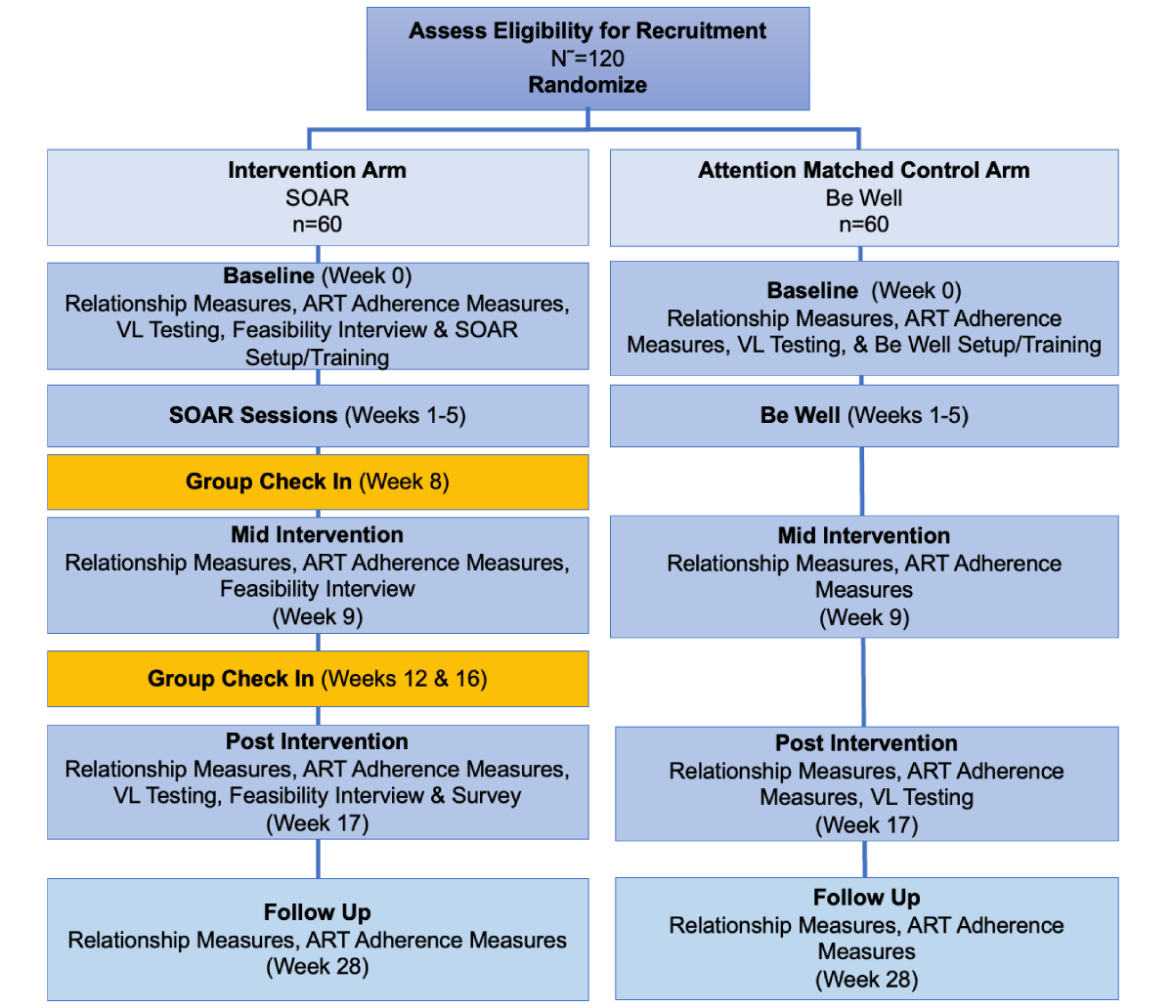
Speaking Out & Allying Relationships (SOAR) Intervention Design for Feasibility Pilot-Testing. ART: antiretroviral treatment; VL: viral load.

SOAR will be assessed by conducting a pilot randomized controlled trial that includes an attention-matched control arm (Figure 1) [40]. Participants will be followed for 28 weeks (baseline, group sessions [weeks 1-5], group check-ins [weeks 8-16], and follow-up [at 28 weeks]).

### Intervention Arm Procedures

#### Group Sessions

Participants will receive the intervention for 5 weeks in 2-hour sessions (1 session per week). Participants will complete the session with the same group of GBMSM. After providing consent, participants will complete Zoom training, which will include a simulated conversation with the interventionist, who will use Zoom in the same room as the participants. Then, each participant’s smartphone will be assessed and set up for compatibility for Zoom. Participants will be notified that they will receive an SMS text message reminder 12 hours before each session. At 1 hour before a session, participants will receive an SMS text message with the Zoom link for that session. Participants will complete several self-assessments and develop an action plan during the intervention. Participants will be provided with the partner referral letters via SMS text messaging (REDCap [Research Electronic Data Capture]; Vanderbilt University) or in paper form (their choice) at the start of the study; multiple copies of the referral letters may be requested throughout the study.

#### Group Check-ins

During the last intervention session, participants will be reminded that they will complete group check-ins (n=3) via Zoom. The dates and times will be provided during the session and then sent via SMS text messages to their mobile phones. Similar to the session procedures, participants will receive an SMS text message reminder 12 hours before a group session and a Zoom link for that session 1 hour before each session.

### Attention-Matched Control Procedures

The control arm will maintain their standard of care and receive information that focuses on topic areas like exercise, nutrition, chronic disease, and sexuality. This content will be based on the South African Ministry of Health B-Wise website (B-Wise) [48]. B-Wise is a general healthy living informational website with significant health content. B-Wise and HR are grounded in similar theoretical frameworks that posit that health information and modeling will empower individuals to make healthy decisions [28,29]. Attention-matched control activities will entail (1) a total of 5 health-related videos that will be sent as Vimeo (Vimeo Inc) web links with passwords via SMS text messaging (1 per week for 5 weeks) and (2) a set of informational-only, 1-way SMS text messages based on B-Wise content (1 per week for 3 months). The videos will correspond with the group sessions, and the 1-way messages will correspond with group check-ins.

### Measures

#### Overview of Measures

Feasibility is the primary outcome for the study, and the secondary outcomes relate to relationship communication, ART adherence, and changes in HIV viral load. Feasibility will be assessed for intervention participants, whereas the secondary outcomes will be assessed for both study arms. Survey measures, interviews, and a biomedical marker of viral load will be used.

#### Feasibility Surveys

There are 4 domains of feasibility—feasibility [49], acceptability [50], willingness [24], and safety [51]. Feasibility is the ability to recruit participants, retain participants, send and receive messages, and participate in group sessions [49]. Acceptability is the degree to which participants like or dislike components of the intervention [52]. Willingness refers to participants’ interest in enrolling in a longer trial and recommending the intervention to others [24]. Safety is the ability to ensure the confidentiality and security of participant data and communication within and outside the intervention [51]. To assess acceptability, the Self-Intervention Evaluation Form and the Client Satisfaction Questionnaire will be administered [53,54]. In addition, a study-specific Likert scale survey will be developed to assess feasibility, willingness, and safety [34]. The study-specific survey will assess participants’ perceived ability to send and receive messages and participate in group sessions and group check-ins (feasibility), the likelihood of participants enrolling in a longer study or referring other GBMSM (willingness), the likelihood of participants providing partners a referral letter (willingness), and perceived intervention confidentiality and security (safety).

#### Feasibility Interviews

In order to understand the feasibility domains, we will conduct 30-minute interviews with purposively selected intervention arm participants (n=30) based on their intervention engagement at baseline, midintervention, and postintervention [55]. Feasibility will be measured by examining participants’ attitudes toward various aspects of the intervention, including video-group interactions; referral letters; and perceived changes in the ability to self-manage ART, HIV risk, and HIV disclosure [56]. Acceptability will be evaluated by examining whether participants like or dislike intervention components and the intervention as a whole [56]. Willingness will be assessed by measuring participants’ willingness to use the intervention from beginning to end, their willingness to use the intervention in different contexts, and their willingness to suggest the intervention to others [56]. Safety will be examined by measuring participants’ perceived levels of discomfort with different components of the intervention and their perceptions of personal safety and unwanted disclosures [51].

#### Relationship, ART Adherence, and Biomedical Measures

Although our study is not powered to detect changes in these areas, the outcomes from these measures will identify potential directions of effect and inform power calculations for a future efficacy trial. Central to the intervention is creating skills for GBMSM living with HIV to talk to their partners about HIV. The key area—relationship satisfaction—will be assessed by using a 10-item scale that assesses satisfaction with both the partner and the relationship [57]. Communication with partners will be assessed by using the short form (11 items) of the Communications Patterns Questionnaire, which assesses communication and conflict resolution [58]. Disclosure will be measured by using both Kalichman and Nachimson’s [59] HIV Disclosure Intent Scale and study-specific questions asking whether the participants disclosed their HIV status to their partners. The questions will also explore participants’ partners’ reactions, the participant-reported partner uptake of a referral letter, and the reported uptake of HIV testing by a partner. HIV treatment adherence will be assessed by using a visual analog scale [60], and HIV viral load testing will be conducted by using dried blood spots. Relationship and ART adherence measures will be administered from baseline through follow-up, and biomedical measures will be administered at baseline and postintervention to all randomized participants.

### Statistical Analysis

Based on our experience from prior studies, we expect around a 23% loss to follow-up, which will result in 92 participants; therefore, we expect to retain 46 participants per group from enrollment through follow-up at 28 weeks. Like other pilot studies, ours is not powered to show the efficacy of the intervention in the study population; the aim of the study is to establish feasibility and the preliminary impact on HIV treatment outcomes before moving to a larger efficacy trial powered for clinical outcomes. We will be able to assess the preliminary impact of SOAR on ART adherence, behavioral measures, and viral load measures. To this end, our sample size (46 participants per group in the randomized controlled trial, yielding data on 92 participants) will yield 70% statistical power with a type 1 error rate at .05 to show a difference between a behavior frequency of 30% in the attention-matched control group and a behavior frequency of 55% in the intervention group for all outcomes.

### Aim 2 Participants

All participants will be (1) GBMSM; (2) those aged ≥18 years; (3) those who have been in a relationship for more than 1 month; (4) those who own a smartphone; (5) those who are comfortable with group discussions about HIV; (6) those living with HIV, as determined via confirmatory testing using OraQuick (OraSure Technologies) during screening; (7) those who live in Eastern Cape province; and (8) those who have been prescribed ART but are suboptimally adherent, as measured by a visual analog scale [60]. Participants will self-report (1) being in a relationship with a man (relationships will be defined as a “person you feel romantically or emotionally connected to above all others, and may be called a partner, boyfriend, lover etc”) for more than 1 month, (2) not having disclosed their HIV status to their partners, and (3) having a partner whose HIV status is negative or unknown. All participants will be recruited and screened into the study by the interventionist. Participants will be randomized 1:1 to either the intervention arm (n=60) or the attention-matched control arm (n=60). All participants will complete written informed consent and receive R150 (around US $10) as a reimbursement for attending study visits.

## Results

Intervention adaptation began in August 2021, and initial tailoring and the refining of GBMSM intervention preferences were completed in December 2021. Usability and feasibility assessments will be completed by March 2022 and February 2024, respectively.

## Discussion

### Study Implications

There are poor HIV treatment outcomes for GBMSM in South Africa, given the high HIV prevalence and low ART adherence rates [3,4], which fuel HIV transmission between partners with limited skills in HIV disclosure and communication [2]. To address these outcomes, our adapted SOAR intervention will help GBMSM in South Africa to build skills for coping with HIV-related stress, disclosing one’s HIV serostatus and sexuality, and improving communication in relationships. We will assess the intervention’s feasibility, its acceptability to participants, its safety, and participants’ willingness to use the intervention.

The innovation of the proposed intervention—SOAR—arises from 4 critical, interconnected knowledge gaps. First, it will address the lack of efficacious interventions that address partnership dynamics and, in turn, affect HIV prevention and treatment outcomes for ART adherence and viral suppression among GBMSM in a highly stigmatized, resource-limited setting in South Africa [2,25]. Second, the study will mitigate gaps in the effective adaptation of evidence-based interventions to address the HIV prevention and treatment outcomes of GBMSM who are pursuing relationships in this setting [61]. Toward this goal, GBMSM’s preferences for HIV prevention (U=U and PrEP knowledge and partner referrals) and HIV treatment (partner disclosure and ART dose planning) domains will be determined and integrated into the intervention. This approach aims to build GBMSM’s self-efficacy in these domains. Further, HCD approaches will be used for adaptation, generating new methods for supporting the increased use of videoconferences for intervention delivery and evidence-based interventions, especially in low-resource communities [47,62]. Third, previous applications of mHealth have focused on SMS text message–delivered HIV prevention and treatment content; aside from the recent work by Essien et al [37] and Henwood et al [36], there are no studies that have harnessed the smartphone capacity of GBMSM in African settings and the commonly used Zoom platform to improve engagement in HIV treatment, which is influenced by relationship dynamics. Finally, our study will address the dearth of HIV research engagement among GBMSM in Eastern Cape province, South Africa, by generating feasibility data to inform a larger clinical trial for measuring the efficacy of the SOAR intervention in effecting viral suppression among GBMSM and improving the referral and uptake of HIV testing and PrEP services among their partners. These are also key to reducing HIV incidence, especially among serodiscordant couples [14,25]. There is a low willingness to use PrEP among partnered GBMSM in South Africa [25], but our partner referral approach may demonstrate feasibility for increasing PrEP uptake among high-risk male couples and may result in the development of PrEP interventions for couples who may be tested in future efficacy trials.

### Conclusion

There is an urgent need to develop interventions that provide GBMSM with the behavioral skills for addressing the management of HIV disease while in relationships. If feasible, the proposed intervention has the potential to be implemented in other sub-Saharan African settings with high HIV prevalence rates among GBMSM. Empowering GBMSM to manage their ART adherence and serostatus disclosure while they build a relationship has the potential to be a low-cost and sustainable mechanism for increasing the uptake of HIV care among GBMSM—a group that is currently overlooked in programmatic efforts in sub-Saharan Africa.

## Abbreviations

ART: antiretroviral treatment
GBMSM: gay, bisexual, and other men who have sex with men
HCD: human-centered design
HR: Healthy Relationships
IT: information technology
mHealth: mobile health
PrEP: pre-exposure prophylaxis
REDCap: Research Electronic Data Capture
SCT: social cognitive theory
SOAR: Speaking Out & Allying Relationships
U=U: Undetectable=Untransmittable

## References

[1] Sanders EJ, Okuku HS, Smith AD, Mwangome M, Wahome E, Fegan G, Peshu N, van der Elst EM, Price MA, McClelland RS, Graham SM (2013). High HIV-1 incidence, correlates of HIV-1 acquisition, and high viral loads following seroconversion among MSM. AIDS.

[2] Daniels J, Struthers H, Maleke K, Lane T, McIntyre J, Coates T (2019). 'My Tablets are on Top of the Fridge': The roles of relationship desire and medical mistrust in ART adherence for HIV-positive MSM and transgender women living in rural South Africa. AIDS Behav.

[3] Lane T, Osmand T, Marr A, Shade SB, Dunkle K, Sandfort T, Struthers H, Kegeles S, McIntyre JA (2014). The Mpumalanga Men's Study (MPMS): results of a baseline biological and behavioral HIV surveillance survey in two MSM communities in South Africa. PLoS One.

[4] Sandfort TGM, Dominguez K, Kayange N, Ogendo A, Panchia R, Chen YQ, Chege W, Cummings V, Guo X, Hamilton EL, Stirratt M, Eshleman SH (2019). HIV testing and the HIV care continuum among sub-Saharan African men who have sex with men and transgender women screened for participation in HPTN 075. PLoS One.

[5] Lippman SA, Lane T, Rabede O, Gilmore H, Chen YH, Mlotshwa N, Maleke K, Marr A, McIntyre JA (2018). High acceptability and increased HIV-testing frequency after introduction of HIV self-testing and network distribution among South African MSM. J Acquir Immune Defic Syndr.

[6] Lane T, Osmand T, Marr A, Struthers H, McIntyre JA, Shade SB (2016). Brief report: High HIV incidence in a South African community of men who have sex with men: Results from the Mpumalanga Men's Study, 2012-2015. J Acquir Immune Defic Syndr.

[7] Duby Z, Nkosi B, Scheibe A, Brown B, Bekker LG (2018). 'Scared of going to the clinic': Contextualising healthcare access for men who have sex with men, female sex workers and people who use drugs in two South African cities. South Afr J HIV Med.

[8] Daniels J, Struthers H, Lane T, Maleke K, McIntyre J, Coates T (2018). "Booze is the main factor that got me where I am today": alcohol use and HIV risk for MSM in rural South Africa. AIDS Care.

[9] Tucker A, de Swardt G, Struthers H, McIntyre J (2013). Understanding the needs of township men who have sex with men (MSM) health outreach workers: exploring the interplay between volunteer training, social capital and critical consciousness. AIDS Behav.

[10] Tucker A, Liht J, de Swardt G, Arendse C, McIntyre J, Struthers H (2016). Efficacy of tailored clinic trainings to improve knowledge of men who have sex with men health needs and reduce homoprejudicial attitudes in South Africa. LGBT Health.

[11] Shaver J, Sullivan P, Siegler A, de Voux A, Phaswana-Mafuya N, Bekker LG, Baral SD, Wirtz AL, Beyrer C, Brown B, Stephenson R (2017). Comparing provider and client preferences for HIV prevention services in South Africa among men who have sex with men. J Int Assoc Provid AIDS Care.

[12] van der Elst EM, Gichuru E, Muraguri N, Musyoki H, Micheni M, Kombo B, Smith AD, Graham SM, Sanders EJ, Operario D (2015). Strengthening healthcare providers' skills to improve HIV services for MSM in Kenya. AIDS.

[13] Eisinger RW, Dieffenbach CW, Fauci AS (2019). HIV viral load and transmissibility of HIV infection: Undetectable equals untransmittable. JAMA.

[14] Kahle EM, Sullivan S, Stephenson R (2018). Functional knowledge of pre-exposure prophylaxis for HIV prevention among participants in a web-based survey of sexually active gay, bisexual, and other men who have sex with men: Cross-sectional study. JMIR Public Health Surveill.

[15] Maleke K, Daniels J, Lane T, Struthers H, McIntyre J, Coates T (2019). How social stigma sustains the HIV treatment gap for MSM in Mpumalanga, South Africa. Glob Health Promot.

[16] Charurat ME, Emmanuel B, Akolo C, Keshinro B, Nowak RG, Kennedy S, Orazulike I, Ake J, Njoku O, Baral S, Blattner W, TRUST Study Group (2015). Uptake of treatment as prevention for HIV and continuum of care among HIV-positive men who have sex with men in Nigeria. J Acquir Immune Defic Syndr.

[17] Graham SM, Micheni M, Kombo B, Van Der Elst EM, Mugo PM, Kivaya E, Aunon F, Kutner B, Sanders EJ, Simoni JM (2015). Development and pilot testing of an intervention to promote care engagement and adherence among HIV-positive Kenyan MSM. AIDS.

[18] Knox J, Reddy V, Kaighobadi F, Nel D, Sandfort T (2013). Communicating HIV status in sexual interactions: assessing social cognitive constructs, situational factors, and individual characteristics among South African MSM. AIDS Behav.

[19] Daniels J, Maleke K, Lane T, Struthers H, McIntyre J, Kegeles S, Moore A, Coates T (2017). Learning to live with HIV in the rural townships: A photovoice study of men who have sex with men living with HIV in Mpumalanga, South Africa. J Assoc Nurses AIDS Care.

[20] Kunzweiler CP, Bailey RC, Okall DO, Graham SM, Mehta SD, Otieno FO (2017). Factors associated with prevalent HIV infection among Kenyan MSM: The Anza Mapema study. J Acquir Immune Defic Syndr.

[21] Daniels J, Lane T, Maleke K, Mogos W, Assaf R, Struthers H, McIntyre J, Coates T (2018). Exploring dual disclosures for men who have sex with men in Mpumalanga, South Africa: a report from the field. Afr J AIDS Res.

[22] Goodreau SM, Carnegie NB, Vittinghoff E, Lama JR, Sanchez J, Grinsztejn B, Koblin BA, Mayer KH, Buchbinder SP (2012). What drives the US and Peruvian HIV epidemics in men who have sex with men (MSM)?. PLoS One.

[23] Sullivan PS, Salazar L, Buchbinder S, Sanchez TH (2009). Estimating the proportion of HIV transmissions from main sex partners among men who have sex with men in five US cities. AIDS.

[24] Stephenson R, Finneran C, Goldenberg T, Coury-Doniger P, Senn TE, Urban M, Schwartz A, Sullivan P (2015). Willingness to use couples HIV testing and discussion of sexual agreements among heterosexuals. Springerplus.

[25] Stephenson R, Darbes LA, Chavanduka T, Essack Z, van Rooyen H (2021). HIV testing, knowledge and willingness to use PrEP among partnered men who have sex with men in South Africa and Namibia. AIDS Behav.

[26] Jewkes R, Gibbs A, Jama-Shai N, Willan S, Misselhorn A, Mushinga M, Washington L, Mbatha N, Skiweyiya Y (2014). Stepping Stones and Creating Futures intervention: shortened interrupted time series evaluation of a behavioural and structural health promotion and violence prevention intervention for young people in informal settlements in Durban, South Africa. BMC Public Health.

[27] Kalichman SC, Rompa D, Cage M, DiFonzo K, Simpson D, Austin J, Luke W, Buckles J, Kyomugisha F, Benotsch E, Pinkerton S, Graham J (2001). Effectiveness of an intervention to reduce HIV transmission risks in HIV-positive people. Am J Prev Med.

[28] Marhefka SL, Iziduh S, Fuhrmann HJ, Lopez B, Glueckauf R, Lynn V, Baldwin J (2013). Internet-based video-group delivery of Healthy Relationships--a "prevention with positives" intervention: report on a single group pilot test among women living with HIV. AIDS Care.

[29] Marhefka SL, Buhi ER, Baldwin J, Chen H, Johnson A, Lynn V, Glueckauf R (2014). Effectiveness of healthy relationships video-group-A videoconferencing group intervention for women living with HIV: preliminary findings from a randomized controlled trial. Telemed J E Health.

[30] Marhefka SL, Fuhrmann HJ, Gilliam P, Lopez B, Baldwin J (2012). Interest in, concerns about, and preferences for potential video-group delivery of an effective behavioral intervention among women living with HIV. AIDS Behav.

[31] (2012). South African mobile generation: Study on South African young people on mobiles. UNICEF.

[32] Smillie K, Van Borek N, van der Kop ML, Lukhwaro A, Li N, Karanja S, Patel AR, Ojakaa D, Lester RT (2014). Mobile health for early retention in HIV care: a qualitative study in Kenya (WelTel Retain). Afr J AIDS Res.

[33] Hirsch-Moverman Y, Daftary A, Yuengling KA, Saito S, Ntoane M, Frederix K, Maama LB, Howard AA (2017). Using mHealth for HIV/TB treatment support in Lesotho: Enhancing patient-provider communication in the START study. J Acquir Immune Defic Syndr.

[34] Bauermeister JA, Tingler RC, Demers M, Harper GW (2017). Development of a tailored HIV prevention intervention for single young men who have sex with men who meet partners online: Protocol for the myDEx project. JMIR Res Protoc.

[35] Dulli L, Ridgeway K, Packer C, Plourde KF, Mumuni T, Idaboh T, Olumide A, Ojengbede O, McCarraher DR (2018). An online support group intervention for adolescents living with HIV in Nigeria: A pre-post test study. JMIR Public Health Surveill.

[36] Henwood R, Patten G, Barnett W, Hwang B, Metcalf C, Hacking D, Wilkinson L (2016). Acceptability and use of a virtual support group for HIV-positive youth in Khayelitsha, Cape Town using the MXit social networking platform. AIDS Care.

[37] Essien EJ, Mgbere O, Monjok E, Ekong E, Holstad MM, Kalichman SC (2011). Effectiveness of a video-based motivational skills-building HIV risk-reduction intervention for female military personnel. Soc Sci Med.

[38] Daniels J, Struthers H, Soler J, Ricco E, Blackmon J, Teklehaimanot S, McIntyre J, Coates T (2021). Building self-advocacy in HIV care: the use of role-play to examine healthcare access for HIV-positive MSM in rural South Africa. Glob Health Promot.

[39] Daniels J, Lane T, Struthers H, Maleke K, Moges W, McIntyre J, Coates T (2017). Assessing the feasibility of smartphone apps for HIV-care research with MSM and transgender individuals in Mpumalanga, South Africa. J Int Assoc Provid AIDS Care.

[40] Jemmott JB 3rd, Jemmott LS, O'Leary A, Ngwane Z, Icard LD, Heeren GA, Mtose X, Carty C (2014). Cluster-randomized controlled trial of an HIV/sexually transmitted infection risk-reduction intervention for South African men. Am J Public Health.

[41] Garofalo R, Kuhns LM, Hotton A, Johnson A, Muldoon A, Rice D (2016). A randomized controlled trial of personalized text message reminders to promote medication adherence among HIV-positive adolescents and young adults. AIDS Behav.

[42] Safren SA, Traeger L, Skeer MR, O'Cleirigh C, Meade CS, Covahey C, Mayer KH (2010). Testing a social-cognitive model of HIV transmission risk behaviors in HIV-infected MSM with and without depression. Health Psychol.

[43] Ingersoll K, Dillingham R, Reynolds G, Hettema J, Freeman J, Hosseinbor S, Winstead-Derlega C (2014). Development of a personalized bidirectional text messaging tool for HIV adherence assessment and intervention among substance abusers. J Subst Abuse Treat.

[44] Lyon AR, Munson SA, Renn BN, Atkins DC, Pullmann MD, Friedman E, Areán PA (2019). Use of human-centered design to improve implementation of evidence-based psychotherapies in low-resource communities: Protocol for studies applying a framework to assess usability. JMIR Res Protoc.

[45] Davey DJ, Wall KM, Serrao C, Prins M, Feinberg M, Mtonjana N, Hlophe K, Zuma L, Sejake S, Malone T (2019). HIV positivity and referral to treatment following testing of partners and children of PLHIV index patients in public sector facilities in South Africa. J Acquir Immune Defic Syndr.

[46] Stephenson R, Bratcher A, Mimiaga MJ, Garofalo R, Hidalgo MA, Hoehnle S, Sullivan PS (2020). Brief report: Accuracy in self-report of viral suppression among HIV-positive men with HIV-negative male partners. J Acquir Immune Defic Syndr.

[47] Harte R, Quinlan LR, Glynn L, Rodríguez-Molinero A, Baker PM, Scharf T, ÓLaighin G (2017). Human-centered design study: Enhancing the usability of a mobile phone app in an integrated falls risk detection system for use by older adult users. JMIR Mhealth Uhealth.

[48] B-Wise. B-Wise.

[49] Wray T, Chan PA, Simpanen E, Operario D (2017). eTEST: Developing a smart home HIV testing kit that enables active, real-time follow-up and referral after testing. JMIR Mhealth Uhealth.

[50] Muessig KE, LeGrand S, Horvath KJ, Bauermeister JA, Hightow-Weidman LB (2017). Recent mobile health interventions to support medication adherence among HIV-positive MSM. Curr Opin HIV AIDS.

[51] Choko AT, Kumwenda MK, Johnson CC, Sakala DW, Chikalipo MC, Fielding K, Chikovore J, Desmond N, Corbett EL (2017). Acceptability of woman-delivered HIV self-testing to the male partner, and additional interventions: a qualitative study of antenatal care participants in Malawi. J Int AIDS Soc.

[52] Zanolini A, Chipungu J, Vinikoor MJ, Bosomprah S, Mafwenko M, Holmes CB, Thirumurthy H (2018). HIV self-testing in Lusaka Province, Zambia: Acceptability, comprehension of testing instructions, and individual preferences for self-test kit distribution in a population-based sample of adolescents and adults. AIDS Res Hum Retroviruses.

[53] Svenningsson I, Petersson EL, Udo C, Westman J, Björkelund C, Wallin L (2019). Process evaluation of a cluster randomised intervention in Swedish primary care: using care managers in collaborative care to improve care quality for patients with depression. BMC Fam Pract.

[54] Larsen DL, Attkisson CC, Hargreaves WA, Nguyen TD (1979). Assessment of client/patient satisfaction: development of a general scale. Eval Program Plann.

[55] Palinkas LA, Horwitz SM, Green CA, Wisdom JP, Duan N, Hoagwood K (2015). Purposeful sampling for qualitative data collection and analysis in mixed method implementation research. Adm Policy Ment Health.

[56] Knight L, Makusha T, Lim J, Peck R, Taegtmeyer M, van Rooyen H (2017). "I think it is right": a qualitative exploration of the acceptability and desired future use of oral swab and finger-prick HIV self-tests by lay users in KwaZulu-Natal, South Africa. BMC Res Notes.

[57] Miller RS, Lefcourt HM (1982). The assessment of social intimacy. J Pers Assess.

[58] Christensen A, Shenk JL (1991). Communication, conflict, and psychological distance in nondistressed, clinic, and divorcing couples. J Consult Clin Psychol.

[59] Kalichman SC, Nachimson D (1999). Self-efficacy and disclosure of HIV-positive serostatus to sex partners. Health Psychol.

[60] Finitsis DJ, Pellowski JA, Huedo-Medina TB, Fox MC, Kalichman SC (2016). Visual analogue scale (VAS) measurement of antiretroviral adherence in people living with HIV (PLWH): a meta-analysis. J Behav Med.

[61] Wingood GM, DiClemente RJ (2008). The ADAPT-ITT model: a novel method of adapting evidence-based HIV interventions. J Acquir Immune Defic Syndr.

[62] Billings DW, Leaf SL, Spencer J, Crenshaw T, Brockington S, Dalal RS (2015). A randomized trial to evaluate the efficacy of a web-based HIV behavioral intervention for high-risk African American women. AIDS Behav.

